# DELAYED DIAGNOSIS IN A CASE OF PERIANAL TUBERCULOSIS: DIFFERENTIAL DIAGNOSIS IN PERIANAL ULCERATION

**DOI:** 10.4103/0019-5154.70702

**Published:** 2010

**Authors:** Guzin Ozarmagan, Sinem Keles, K Didem Yazganoglu, Necmettin Sokucu

**Affiliations:** *From the Department of Dermatology Turkey. E-mail: karadidem@yahoo.com*; 1 *From the Department of General Surgery, Istanbul Medical Faculty, Istanbul University, Turkey*

Periorificial tuberculosis is a rare form of extrapulmonary tuberculosis.[1–4] Diagnosis and treatment can be delayed in this type of tuberculosis as the differential diagnosis includes a large spectrum of diseases. An asymptomatic pulmonary tuberculosis case presenting with perianal ulceration, initially mistaken for a herpetic ulcer and Crohn’s disease, is reported here.

**Figure 1:**
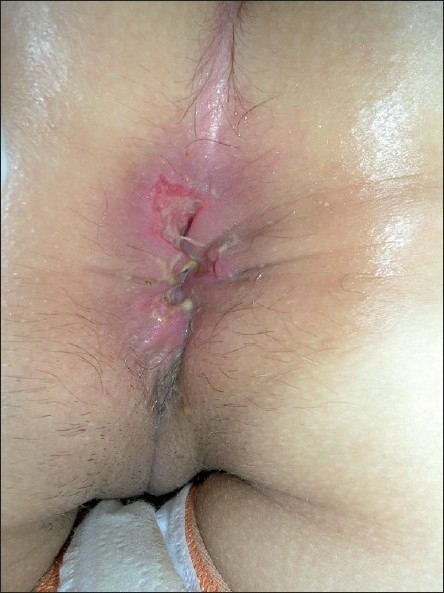
Superficial perianal ulcerations at admission

A 28 year-old woman presented with a three-month history of two superficial, painful perianal ulcerations [Figure 1] with no inguinal lymphadenopathy accompanied. While colonoscopy was normal, biopsy showed nonspecific ulceration and granulation tissue rich with plasma cells. Cytomegalovirus (CMV), herpes simplex virus (HSV), human immunodeficiency virus (HIV), syphilis, hepatitis B and C serology were negative. Polymerase chain reaction from the tissue material was positive for HSV-1 and negative for HSV-2. She had hypochromic microcytic anemia and a high erythrocyte sedimentation rate (36 mm/h).

Initial diagnosis was genital herpes, but valacyclovir treatment did not heal the ulcerations. Another biopsy revealed granulomatous infiltration with Langhans type multinuclear giant cells, lymphocytes and histiocytes without necrosis. As necrosis was not observed with granulomatous infiltration and Ziehl-Neelsen stain did not show any acid-fast bacilli (AFB), Crohn’s disease was primarily considered in the diagnosis. Two-week therapy with methylprednisolone and cyclosporine worsened the ulcerations, fever and night sweats were added to the complaints. Therefore therapy was stopped. Tuberculosis was considered in the diagnosis. Computed tomography of thorax showed bilateral miliary and cavitary infiltration on the right upper lobar posterior segment. The patient was anergic to the Mantoux test. An aspiration fluid specimen examined for AFB was negative with Ziehl-Neelsen stain, but positive with culture for *Mycobacterium tuberculosis (M. tuberculosis)*.

The final diagnosis was pulmonary miliary tuberculosis with perianal tuberculosis. In the fifteenth day of four regimen antituberculosis therapy, perianal ulceration and pulmonary symptoms resolved.

Skin involvement is a rare form of extrapulmonary tuberculosis, whereas perianal tuberculosis representing a form of cutaneous tuberculosis, is even more rare.[1–4] Periorificial tuberculosis results from autoinoculation of *M. tuberculosis* in patients with pulmonary, intestinal or genitourinary tuberculosis and manifests as oral, genital or anal ulcerations.[1] The bacilli are thought to reach and attack the traumatized mucosa or skin in swallowed sputum in cases of pulmonary tuberculosis.[2,3] Rarely, it can occur as a result of hematogenous, lymphatic or direct spread of the disease.[2,3] The initial manifestation of pulmonary tuberculosis can be these ulcers as also observed in our case.[1] Perianal tuberculosis is also reported without any presence of gastrointestinal or pulmonary tuberculosis.[4] Tuberculin test can be negative in these patients.[1,3] Our patient was also anergic, which can be attributed to the miliary tuberculosis or immunosuppressive therapy.

Clinically indistinguishable from other causes of perianal ulcers, the differential diagnosis consists infectious diseases like syphilis, lymphogranuloma venereum, granuloma inguinale, chancroid, leishmaniasis, deep mycoses, amoebiasis; infections with HSV, CMV, HIV, varicella zoster virus, *Cryptococcus neoformans, Mycobacterium avium/intracellulare*; inflammatory bowel diseases like Crohn’s disease, ulcerative colitis; pyoderma gangrenosum; sarcoidosis; neoplasias; and trauma.[1–5]

In conclusion, perianal ulcers can be the initial manifestation of tuberculosis even in asymptomatic patients. In order to make an early, exact diagnosis and start an appropriate treatment, in addition to histopathological examination, AFB must be searched both with Ziehl-Neelsen stain and culture.
